# High incidence rate of postoperative sore throat in intubated children at Northwest Amhara Comprehensive Specialized Hospitals, Ethiopia. A multicenter study

**DOI:** 10.3389/fped.2023.1037238

**Published:** 2023-03-01

**Authors:** Misganaw Terefe Molla, Yosef Belay Bizuneh, Yonas Addisu Nigatu, Debas Yaregal Melesse

**Affiliations:** ^1^ Department of Anesthesia, College of Medicine and Health Sciences, Bahirdar University, Bahirdar, Ethiopia; ^2^ Department of Anesthesia, College of Medicine and Health Sciences, University of Gondar, Gondar, Ethiopia

**Keywords:** children, anesthesia complications, intubation, postoperative, sore throat

## Abstract

**Background:**

Postoperative sore throat is the most frequent complication in pediatric patients after general anesthesia. This study aimed to investigate the incidence of postoperative sore throat in patients undergoing general anesthesia with tracheal intubation or laryngeal mask airway.

**Methods:**

A hospital-based multicenter prospective observational cohort study was conducted. Proportional allocation was done with a total of 424 patients from March 1 to June 30, 2022. The information was entered into the Epi-Data software version 4.6 and analyzed with Stata 14. Socio–demographic, surgical, and anesthetic-related characteristics were analyzed using descriptive statistics. A *p*-value of less than 0.2 was the cutpoint of bivariate logistic regression analysis, and *p*-values of less than 0.05 were regarded as statistically significant in multivariate logistic regression to determine the presence and strength of association between independent variables and postoperative sore throat.

**Results:**

A total of 411 patients were included in this study, with a response rate of 96.9%. The overall proportion of patients who developed postoperative sore throat was 45% (95% CI: 40.18–49.84). Patients who had anesthesia for more than two hours (AOR = 8.23: 95% CI = 4.08–16.5), those who were intubated by undergraduate anesthesia students (AOR = 2.67: 95% CI = 1.53–4.67), and those who had been intubated using tracheal tube (AOR = 2.38: 95% CI = 1.15–4.92) were significantly associated with the level of postoperative sore throat.

**Conclusions and recommendations:**

We concluded that intubated children with ETT have a high incidence of post-operative sore throat. Tracheal tube usage, intubation by undergraduate students, and more than two hours of anesthesia duration were associated factors. The incidence of sore throat can be decreased with the use of a laryngeal mask airway, intubation by a senior anesthetist, and shortening of anesthesia time.

## 1. Introduction

General anesthesia is the most commonly used type of anesthesia for a surgical procedure involving an ETT or LMA for the maintenance of airway patency, resulting in postoperative complications (1–3).

A postoperative sore throat is a pain or discomfort in the patient's throat after receiving general anesthesia under tracheal tube or laryngeal mask airway (4–6). In the pediatric population, the incidence of postoperative sore throat following general anesthesia is not well understood and is thought to occur in 5%–41% of children with tracheal intubation (3, 7–11) and post-operative sore throat is also reported to be lower at 4%–13% in LMA use (12–16). In adult patients, up to 12%–60% get postoperative sore throat, which is a common and undesirable problem (6, 17, 18).

The multiple factors that contribute to post-operative sore throat include the choice of airway device, high tracheal tube cuff pressure, surgical manipulation of the airway, varying circuit humidification, airway suctioning, dehydration, and the type and length of anesthesia and surgery (7, 8, 19–21).

The exact time at which POST can occur is not clearly understood in the pediatric age group, even if some studies state that it occurs as early as 15 min after immediate postoperative time and reduces its incidence from 12 to 24 h (22, 23). However, the duration may continue after discharge, affecting patients' oral intake due to postoperative throat pain (8, 24).

Untreated POST will result in a longer discharge time, a negative experience with anesthesia, a longer postoperative recovery time, severe patient discomfort, distress, anxiety, dissatisfaction, increased hoarseness, and a reduction in patient life activity (3, 25–29).

The preventive and management techniques for POST in pediatric patients are essential for the early reduction of severe complications, including reduction of stimulation, use of the small-sized tube, reducing the number of attempts, use of a non–steroidal anti–inflammatory drug, lidocaine, nebulized magnesium and preemptive ketamine and steroids (30–34).

Postoperative pediatric sore throat is not extensively studied in sub-Saharan countries, even if the problem is significant. Therefore, we aimed to assess the incidence and associated factors of postoperative sore throat among pediatric patients in the Northwest Amhara Referral Hospitals, Ethiopia.

## 2. Methods

### 2.1. Study design, setting and period

A multicenter prospective observational cohort study was conducted. The study was conducted at Northwest Amhara comprehensive specialized hospitals, Ethiopia, from March 1 to June 30, 2022. The study took place at the University of Gondar Comprehensive Specialized Hospital, which has seven general surgery rooms; two obstetrics; two gynecology; and one ophthalmology operation room. Tibebe Ghion Specialized Hospital has seven general surgery rooms; two obstetrics and gynecology; two orthopedic operation rooms; and one ophthalmology operation room. Felege Hiwot Comprehensive Specialized Hospital has six general surgery rooms; two orthopedic; two obstetrics and gynecology rooms; and one ophthalmology operation room. The Debre Tabor Comprehensive Specialized Hospital has three general surgery rooms; two orthopedics; two obstetrics and gynecology operation rooms; and one ophthalmology operation room. The Debre Markos Comprehensive Specialized Hospital has three general surgery rooms; one orthopedic; two obstetrics and gynecology operation rooms; and one ophthalmology operation room. There is no distinct pediatric operating room in the above referral institutions.

### Anaesthesia

Anaesthetic management was standardized. Standard patient monitoring, including an electrocardiogram, end-tidal carbon dioxide, pulse oximetry, and non-invasive arterial blood pressure, was performed. Because the study was cross-sectional and not controlled, anesthesia induction techniques were dependent on patient condition and anesthetist experience level. The cuff was inflated manually with air to a clinical endpoint of loss of an audible leak. The intra-cuff pressure was not measured because a hand-held manometer is not available in our setup.

The age, sex, weight, and physical status of the patients were recorded on a standardized form. Routine informed consent for surgery and anesthesia was obtained before the patient's arrival in the operating room. The type and duration of surgery, the intraoperative airway device used [Endotracheal tube (ETT), Laryngeal Mask Airway (LMA)], the grade of intubation, and the patient's position during surgery were noted. After the end of surgery, the airway devices were removed when patients were able to respond to commands. The patients were taken to the post-anesthesia care unit, from which they were shifted to the ward after adequate recovery.

### 2.2. Study population, inclusion and exclusion criteria and study variables

All pediatric surgical patients admitted at Comprehensive Specialized Hospitals of Northwest Amhara, Ethiopia, from March 1 to June 30, 2022, were considered the source population, and all pediatric surgical patients operated under general anesthesia with ETT or LMA whose age was 6–18 years were considered the study population.

All pediatric surgical patients, both elective and emergency procedures, aged 6–18 years who were operated on under general anesthesia with ETT or LMA within the study period were included in the study, whereas surgical procedures in oral, nasal, and throat surgery; recent respiratory infection; recent preoperative throat pain; postoperative need for a mechanical ventilator; and intensive care were excluded from the study.

The incidence of postoperative sore throat was the primary outcome variable, and the level of severity and associated factors of postoperative sore throat were the secondary outcome variables.

### 2.3. Sample size determination

The sample size was determined by applying a *p*-value of 50% to a single proportion formula. The sample size was calculated with a 95% confidence interval (CI) and a 5% margin of error. Because no other study with the same sociodemographic and study population characteristics has been carried out. So

$$\frac{n=z^{2}(\;pq)}{d^{2}}$$

$$\frac{n=1.96^{2}{}^{*}0.5{}^{*}0.5}{0.05{}^{2}}$$

Then *n* = 385. Where (*p*) represent the estimated proportion, (*z*) level of confidence, (*d*) margin of error, and *n* = desired sample size. Using 385 plus a 10% non-response rate of 39, the total sample size was calculated to be *n* = 424.

### 2.4. Sampling procedure

A multi-center consecutive sampling method was used until the target sample was fully obtained. Additionally, we distributed the sample within each institution using the proportionate allocation approach. We determined the final sample in each hospital by multiplying the total number of calculated sample sizes by the number of patients operated on in the last four months in each hospital, divided by the total number of cases operated in five hospitals. The final number of samples used during the analysis was 411, and because of incomplete data, 13 patients were removed.

### 2.5. The assessment methods and questionnaire

Self-reporting of pain is the ideal method for children who are able to do so. The pain assessment process may also include parents or guardians because their pain ratings are closely related to those of their children. The direct self-report technique can be used to evaluate postoperative patient complaints to help identify their magnitude and contributing factors. A child's self-reported throat pain was graded on a four-point severity scale, and the existence or absence of a sore throat was evaluated by a Yes/No response in an observational cohort study. The pain scale was greater than or equal to 1, and if there was no postoperative throat pain, it was rated as No on a four-point categorical scale with 0 denoting no throat pain, 1 denoting mild throat pain (complain when asked), 2 denoting moderate throat pain (complain of throat pain by itself), and 3 denoting severe throat pain (including change in voice or hoarseness).

### 2.6. Operational definitions

**Junior anesthetist:** BSC holder *and* senior anesthesist: MSc holder
**Pediatric patients:** Age range from 6 years to 18 years (35–37)

To determine whether POST is present or absent, the category pain scale is used. The absence of a sore throat was reported as no, whereas mild, moderate, and severe sore throats were rated as yes (35, 36, 38).

### 2.7. Data collection procedures

The questionnaire and checklist were adapted through the review of different previous similar studies (9, 23, 39–41). The questionnaire was initially prepared in English, then translated to Amharic, and then translated back into English three times to check for consistency. A structured questionnaire was used to collect the data through a face-to-face interview and a checklist for reviewing client charts. The Amharic version of the questionnaire was used for data collection.

The data was gathered by five trained BSC anesthetists, and one senior anesthetist was working as a supervisor. An interview and a review of the records are both steps in the data collection process. The data collection method used for POST was the direct self-report method. It was collected in the post-anesthesia care unit and patient wards. The presence or absence of a sore throat was noted at intervals of two hours, six hours, and twenty-four hours. Additionally, the degree of pain was assessed and classified as mild, moderate, and severe. Patients with both elective and emergency procedures were included. A record review that included information on the patient, anesthesia, and surgical procedures was also conducted in addition to the data collector interview. The questionnaires and checklists were filled out completely and collected daily after checking the completeness and consistency of the data.

### 2.8. Data quality assurance

A pretest at UOGCSH was conducted to assess the questionnaire's clarity, language accent, and grammatical corrections. 22 patients were included in this pretest. To maintain the quality of the data, the data collectors and supervisors were trained for 3 days on the objective of the study, the content of the questionnaire, how to fill out the questionnaire, respondent rights, informed consent, and interview technique, as well as how to maintain the confidentiality and privacy of the study subjects. The principal investigators and supervisors gave feedback and corrections daily to the data collectors. The data were cleaned, coded, and imported into EpiData version 4.6 before being exported to Stata version 14.

### 2.9. Data processing and analysis

Each completed questionnaire was coded on a pre-arranged coding sheet by the principal investigator to minimize errors. Data was entered into a computer using EpiData version 4.6 and exported to Stata version 14 for additional cleaning and analysis. Sorting and cross-tabulation were used to clean the data. Multicollinearity was examined using the variance inflation factor (VIF), which had a value of 1.17. Using the Shapiro-Wilk test, normality was examined for continuous variables. A Hosmer and Lemeshow test was conducted to evaluate the goodness of fit of the binary logistic regression model, and a *p*-value of 0.2 was obtained. The findings were presented according to the variables through text, tables, and graphs.

Initially, bivariable logistic regression analysis was performed between the dependent variable and each of the independent variables. Then all variables with a *p*-value <0.2 from the bivariable logistic regression analysis were fitted into the multivariable logistic regression model to control for possible confounders. An adjusted odds ratio (AOR) with a 95% confidence interval (CI) was used to measure the strength and significance of the association. A *p*-value <0.05 also indicated the presence of a statistically significant association between postoperative sore throat and independent variables.

## 3. Results

### 3.1. Socio-demographic characteristics of the respondents

A total of 411 pediatric patients participated in this study, with an overall response rate of 96.9%. In this study, the median age was 10 years [interquartile range (IQR): 7–13]. 52.6% of the total participants were male. 81.8% of the pediatric patients had an ASA I. Preoperative NPO status for the patient was 56% for less than or equal to 8 h. 68.9% of patients had scheduled operations on an elective basis. Abdominal and urological operations accounted for the majority of the surgical patients (54.7%) (Table 1).

**Table 1:** Patients sociodemographic and surgery-related characteristics of pediatric patients at Northwest Amhara Referral Hospitals, from March 1 to June 30, 2022 (*N* = 411).

Variable	Postoperative sore throat, *n* (%)		Total (*n* = 411)
	Yes	No	
**Sex**			
Male	97 (44.9%)	119 (55.1%)	216 (52.6%)
Female	88 (45.1)	107 (54.9%)	195 (47.4%)
**Postoperative NPO**			
≤8 h	117 (50.9%)	117 (49.1%)	230 (55.9%)
>8 h	72 (39.8%)	109 (60.2%)	181 (44.1%)*
**PONV**			
No PONV	90 (41.5%)	127 (58.5%)	217 (52.8%)
Nausea	16 (41.0%)	23 (59.0%)	39 (9.5%)
Vomiting	45 (52.9%)	40 (47.1%)	85 (20.7%)
Both PONV	34 (48.6%)	36 (51.4%)	70 (17.0%)
**NGT**			
Yes	23 (60.5%)	15 (39.5%)	38 (9.2%)*
No	162 (43.4%)	211 (56.6%)	373 (90.8%)

NPO, Nil per Os; *n* (%), Frequency and Percentage; NGT, Naso Gastric Tube; PONV, Postoperative Nausea and Vomiting.

* Met *χ*^2^ assumption with *p*-value <0.05.

At two hours after surgery, the postoperative surgical pain level assessment revealed that 26.3% of patients reported no pain, 43.8% reported mild pain, 23.1% reported moderate pain, and 6.8% reported severe pain.

At six hours, 15.3% reported no pain, 37.0% reported moderate discomfort, 44.7% mild pain, and 3.0% reported moderate to severe pain.

At 24 hours, 62.8% of patients reported no pain, while 33.9%, 4.1%, and 0.2%, respectively, reported mild, moderate, and severe pain.

### 3.2. Anesthesia-related characteristics of pediatric patients

Among 411 patients, 84.9% of pediatric patients underwent ETT intubation, while all remaining patients underwent LMA. 94.2% of patients had intubation performed with a grade I laryngoscopy view. All patients under ETT intubation had cuffed tubes. During the perioperative period, airways are not used in 88.1% of patients. The median tube size was 5 centimeters [interquartile range (IQR) 4.5–5.5] (Table 2).

**Table 2:** Anesthesia-related variables of pediatric patients at northwest amhara referral hospitals, from march 1 to June 30, 2022 (*N* = 411).

Variables	Postoperative sore throat, *n* (%)		Total (*N* = 411)
	Yes	No	
Education level of anesthetist			
Master	42 (31.3%)	92 (68.7%)	134 (32.6%)
BSc	42 (36.8%)	72 (63.2%)	114 (27.7%)
Undergraduate student	101 (62.0%)	62 (38.0%)	163 (39.7%)*
Tube type			
ETT	172 (49.3%)	177 (50.7%)	349 (84.9%)*
LMA	13 (21%)	49 (79%)	62 (15.1%)
NMB			
Yes	160 (46.4%)	185 (53.6%)	345 (83.9%)
No	25 (37.9%)	41 (62.1%)	66 (16.1%)

NMB, Neuromuscular Blocker; *N* (%), Frequency and Percentage; ETT, tracheal intubation Tube; LMA, Laryngeal Mask Airway; BSc, Bachler of Science.

* Met *χ*^2^ assumption with *p*-value <0.05.

Regarding the induction of anesthesia, 44.5% of patients were induced with ketamine, 21.7% of patients were induced with propofol, 4.1% of patients were induced with thiopental, and 29.7% of patients were induced with both ketamine and propofol. In terms of analgesia, 91.7% of patients received an opioid, 1.7% received diclofenac, 6.1% received paracetamol, and 0.5% received ketamine analgesia. Steroid (dexamethasone) was administered to 98% of the patients.

### 3.3. Incidence of postoperative sore throat, in each hospital, and the occurrence in each time of observation among pediatric patients

The overall proportion of patients who developed postoperative sore throat was 45% (95% CI: 40.18–49.84). The incidence of POST was included in a patient who underwent general anesthesia with ETT or LMA up to 24 h (Table 3).

**Table 3:** The distribution of patients in each hospital in frequency and percentage at Northwest Amhara Referral Hospitals, from March 1 to June 30, 2022(*N* = 411).

Hospital	Postoperative sore throat, *N* (%)		Total (*N* = 411)
	Yes	No	
UOGCSH	39 (45.9%)	46 (54.1%)	85 (20.7%)
TGCSH	45 (45.5%)	54 (54.5%)	99 (24.1%)
FHCSH	31 (34.8%)	58 (65.2%)	89 (21.7%)
DTCSH	38 (55.9%)	30 (44.1%)	68 (16.5%)
DMCSH	32 (45.7%)	38 (54.3%)	70 (17.0%)

*N* (%), Frequency and Percentage.

DMCSH, Debre Markos Comprehensive Specialized Hospital; DTCSH, Debre Tabor Comprehensive Specialized Hospital; FHCSH, Felege Hiwot Comprehensive Specialized Hospital; TGCSH, Tibebe Ghion Comprehensive Specialized Hospital; UOGCSH, University of Gondar Comprehensive Specialized Hospital.

**Figure 1:**
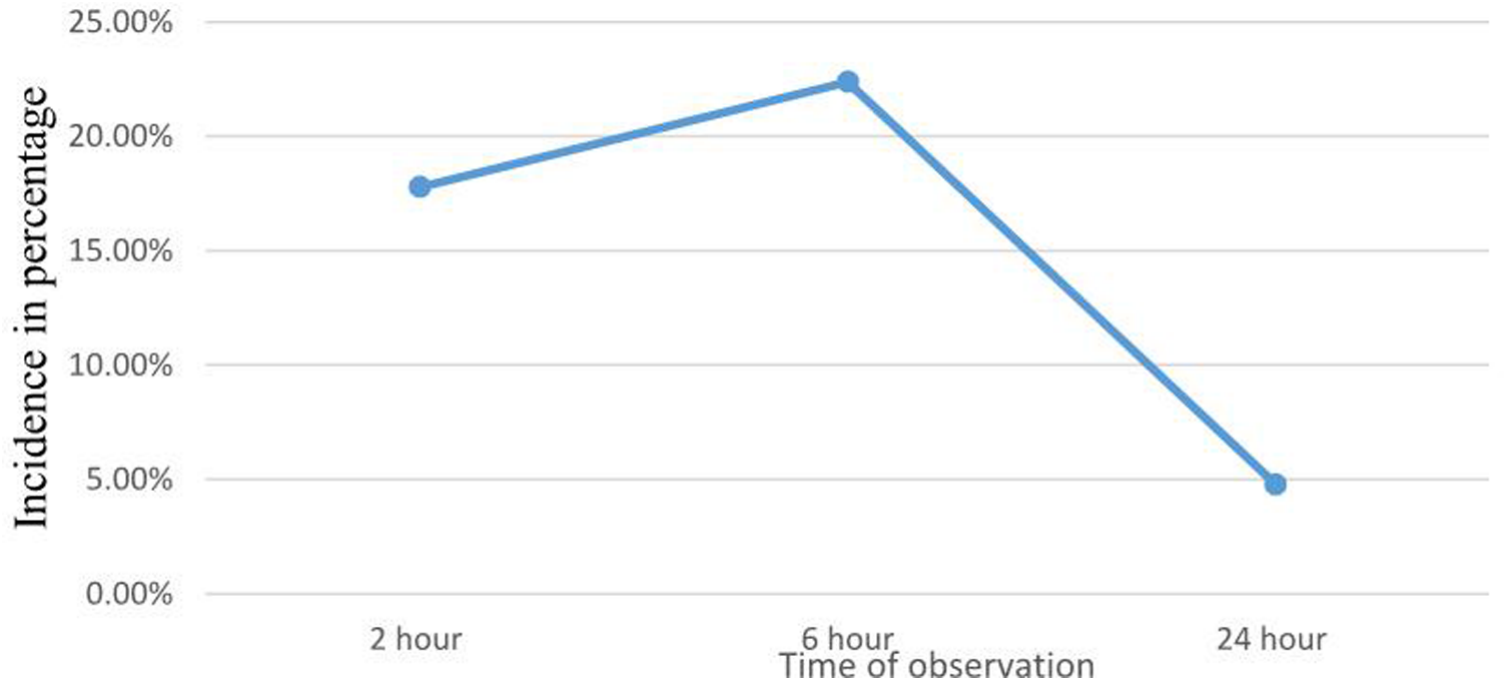
Incidence of POST in each time of observation among pediatric patients at Northwest Amhara Referral Hospitals, from March 1 to June 30, 2022 (*N* = 411).

POST occurred in 17.8% of patients at two hours, with mild, moderate, and severe cases accounting for 79.5%, 12.3%, and 8.2% of the cases, respectively. The incidence of POST at 6 h was 22.4%, and mild, moderate, and severe cases made up 58.7%, 30.4%, and 10.9%, respectively. At 24 h, the incidence was 4.8%, with mild and moderate cases accounting for 80.0% and 20.0%, respectively (Figure 1).

### 3.4. Factors associated with postoperative sore throat among pediatric surgical patients

#### 3.4.1. Bi-variable analysis

In the bi-variate logistic regression analysis, variables with a *p*-value of <0.2 were considered to be factors associated with postoperative sore throat among pediatric surgical patients. These variables included the educational level of the anesthetists who intubated the patients, the tube type (ETT vs. LMA), the presence of NGT, the length of anesthesia, and the postoperative NPO time longer than 8 h (Table 4).

**Table 4:** Bivariate and multivariate logistic regression for POST among pediatric patients at northwest amhara referral hospitals, from march 1 to June 30, 2022(*N* = 411).

Variable	Postoperative sore throat, *n* (%)		COR (95% CI)	AOR (95% CI)	*p*-value
	Yes	No			
**Education**					
Master	42 (31.3%)	92 (68.7%)	1	1	
BSc	42 (36.8%)	72(63.2%)	1.28(0.75–2.16)	1.06(0.58–1.94)	0.85**
Undergraduate	101(62%)	62 (38%)	3.57 (2.2–5.78)	2.67 (1.53–4.67)	0.001*
**Students**					
Tube					
LMA	13 (21%)	49 (79%)	1	1	
ETT	172 (49.3%)	177 (50.7%)	3.66 (1.92–6.99)	2.38 (1.15–4.92)	0.019*
**Duration of Anesthesia**					
<1 h	25 (29.7%)	59 (70.3%)	1	1	
1–2 h	56 (27.8%)	145 (72.2%)	0.91 (0.52–1.59)	0.78 (0.43–1.41)	0.41**
≥2 h	104 (82.5%)	22 (17.5%)	11.2 (5.79–21.5)	8.23 (4.08–16.5)	<0.001*
**NGT**					
No	162 (43.4%)	211 (56.6%)	1	1	
Yes	23 (60.5%)	15 (39.5%)	2.27 (1.24–4.17)	0.99 (0.46–2.11)	0.98**
**Postoperative NPO**					
≤8 h	53 (37.3%)	89 (62.7%)	1	1	
>8 h	132 (49.1%)	137 (50.9%)	1.62 (1.07–2.45)	0.94 (0.56–1.56)	0.79**

*n* (%)= Frequency and Percentage.

COR, Crude Odds Ratio; AOR, Adjusted Odds Ratio; CI, Confidence Interval; ETT, Endotracheal Tube; LMA, laryngeal Mask Airway; NGT, Nasogastric Tube; NPO, Nil Per Os; BSc, Bachler of Science.

* Statistical Significant.

** Statistical Insignificant 1 = Reference Variable.

#### 3.4.2. Multi-variable analysis

In multivariable logistic analysis, a *p*-value of <0.05 was considered statistically significant. The tracheal intubation, the length of anesthesia, and the education level of the anesthetists were factors associated with postoperative sore throat.

Anesthesia lasting more than two hours was 8.23 times more likely to occur POST compared with less than two hours, with 82.5% (AOR = 8.23; CI = 4.08–16.5). Using ETT was 2.38 times more likely to occur POST in comparison to using LMA, with an occurrence of 49.3% (AOR = 2.38; CI = 1.15–4.92). Intubations performed by undergraduate students were 2.67 times more likely to result in POST (62%) than those by junior and senior anesthetists (AOR: 2.67, CI = 1.53–4.67) (Table 4).

## 4. Discussion

Children who undergo ETT or LMA under general anesthesia frequently experience a postoperative sore throat. To the best of our knowledge, no published study has assessed the incidence of POST and its risk factors in pediatric patients in Ethiopia. If prevention and treatment strategies are not implemented during the perioperative phase, postoperative sore throat increases postoperative patient dissatisfaction, anxiety and decreasing the postoperative quality of recovery (42). The main focus of this study was to assess the incidence and associated factors of POST in pediatric surgical patients after general anesthesia ETT vs. LMA.

In this study, the incidence of POST among children after surgery was 45%, with a 95% confidence interval (CI) of (40.1–49.84). This finding is in line with studies conducted in the UK and Korea that showed 41% and 49.5%, respectively (35, 43). The possible explanation might be due to a similar data collection technique. In this study, the incidence of POST among pediatric patients was higher as compared to studies conducted in the UK and Australia, with an incidence of 36.5% and 22.6%, respectively (8, 9). The possible reasons for this difference might be sample size and the study population's socio–demographic characteristics. The incidence of POST was assessed in this study at 2, 6, and 24 h, with a higher incidence at 6 h than at 2 and 24 h. The majority of the patients experience mild POST, followed by moderate and severe. This finding is supported by a study conducted in Pakistan (36), the UK (8), and Korea (35).

POST in pediatric patients is affected by different variables. Among the factors that displayed a statistically significant link in the multivariate logistic regression were patients intubated by undergraduate students, duration of anesthesia greater than 2 h, and use of ETT for intubation (Table 4).

In this study, anesthesia lasting longer than two hours was a statistically significant factor in the occurrence of POST. Anesthesia lasting more than two hours was 8.23 times more likely to result in POST than less than two hours, with an incidence rate of 82.5% (AOR = 8.23; CI = 4.08–16.5). This finding was supported by a study conducted in the UK that showed a longer duration of anesthesia can cause POST with an occurrence of 55.2% (8). The possible explanation for this finding, in general, is that the longer the tracheal tube was in place, the longer the anesthesia lasted, and this caused irritation, inflammation, and ischemia to the airway structure and postoperative dehydration (22). Therefore, keep in mind that shortening the process can help to reduce complications.

In this study, there was a statistically significant association between the use of ETT and POST in pediatric patients when compared to the use of LMA. Using ETT was 2.38 times more likely to occur POST in comparison to using LMA, with an occurrence of 49.3% (AOR = 2.38; CI = 1.15–4.92). This finding was supported by a study conducted in the UK (8), and Australia (9), with 54.7%, and (19%) respectively. In contrast to ETT, POST was less likely to develop when LMA was used, even though it continues to have a role in the development of POST. In our study, 84.9% of patients underwent general anesthesia for surgery utilizing an ETT rather than using an LMA (15.1%). The idea that ETT use on a patient's airway or trachea could cause structural injury, airway mucosal inflammation, and congestion in addition to activating pain receptors in the trachea, which may result in POST, accounts for this finding (44, 45). As a result, even though ETT is typically employed, smooth intubation and extubation may reduce the risk of POST.

Intubation by undergraduate anesthesia students was significantly related to POST and was 2.67 times more likely to result in POST (62%) than those by junior and senior anesthetists (AOR 2.67, CI 1.53–4.67). This finding, which is supported by a study conducted in Switzerland, showed that complications were more likely to emerge when intubation was carried out by lower training individuals, with a 1.7-fold increased risk of postoperative complications (46). Multiple stimulations and attempts during tube insertion and extubation time could be the causes. Therefore, by being aware of the triggering circumstances, the likelihood of complications during anesthesia practice may be decreased.

The use of NGT and prolonged postoperative NPO duration were not significantly associated during multivariate logistic regression analysis in this study, with an AOR of 0.99 (CI = 0.46–2.11) and an AOR of 0.94 (CI = 0.56–1.56), respectively. An investigation carried out in the UK supported this prolonged postoperative NPO period and was not associated with POST in pediatric patients (8). Furthermore, the adult study discovered that the use of NGTs is a significant risk factor for the development of POST in the United Kingdom (47) and Ethiopia (48). However, research on pediatric patients found no conclusive correlation.

Due to the lack of a cuff manometer, we were unable to determine whether or not there was a significant correlation between cuff pressure and POST in this study. However, a previous study on cuff pressure revealed that cuff pressure greater than 20 cmH2O was a predictor of the occurrence of POST (9, 49). This can be brought on by increased airway pressure, which might result in ischemia, pressure sores, and the onset of throat pain (50). Cuffed ETT was not statistically linked in our study. However, a study using un–cuffed ETT demonstrates that it is a risk factor for the emergence of POST (9). The justification offered was that using an un–cuffed one causes an increase in the rate of reintubation, repeated stimulation, and mucosal injury (51). In our study, there was no correlation between POST and patients who underwent orotracheal intubation and we did not compare with nasotracheal intubation, because there were no patients who underwent nasotracheal intubation. However, a Canadian study found that nasotracheal intubation is a risk factor for the development of POST (22).

## 5. Strength and limitations of the study

### 5.1. Strength

According to our research, this is the first study of its kind to be conducted in Ethiopia. It also lays the groundwork for more research. Sufficient sample sizes were used in the investigation, which comprised multiple study centers.

### 5.2. Limitations

All pediatric age groups were not addressed in the study. Response bias may emerge as a result of the use of self-report data gathering techniques. The suctioning and extubation techniques as well as number of attempt to intubate were not evaluated. Due to the lack of a cuff manometer, we were unable to determine ETT cuff pressure.

## 6. Conclusions

Post-operative sore throat was common among pediatric patients in the study areas within the first 24 h following anesthesia. Significant risk factors for postoperative sore throat in pediatric patients after surgery and anesthesia were longer than two hours of anesthesia, ETT intubation, and intubation with undergraduate anesthesia students.

## 7. Recommendations

**To the anesthetists**: We recommend anesthetists use LMA when appropriate, reducing anesthesia time wasted during surgery in one or more ways. Remove the tube early, and put more of a priority on training for atraumatic intubation attempts. High-risk patients are better intubated by senior anesthetists. Based on other study findings, it is better to use airway blunt medication to reduce postoperative sore throat.

**To the researchers:** We encourage researchers to carry out randomized control trials based on ETT vs. LMA and awake vs. deep extubation. The effect of tracheal intubation cuff pressure on postoperative sore throat by the use of a cuff manometer.

## Data Availability

The raw data supporting the conclusions of this article will be made available by the authors, without undue reservation.
